# Two lichens differing in element concentrations have similar spatial patterns of element concentrations responding to road traffic and soil input

**DOI:** 10.1038/s41598-020-76099-x

**Published:** 2020-11-04

**Authors:** Yuan-yuan Wu, Jing Gao, Guo-zhan Zhang, Run-kang Zhao, Ai-qin Liu, Lian-wei Sun, Xing Li, Hong-liang Tang, Liang-cheng Zhao, Xiu-ping Guo, Hua-jie Liu

**Affiliations:** 1 School of Life Sciences, Institute of Life Science and Green Development, Hebei University, Baoding, 071002 Hebei China; 2 Hebei Research Center for Geoanalysis, Baoding, 071051 Hebei China; 3 Hebei Baoding Municipal Drainage Corporation, Baoding, 071051 Hebei China

**Keywords:** Element cycles, Atmospheric chemistry, Environmental monitoring

## Abstract

Two epiphytic lichens (*Xanthoria alfredii*, *XAa*; *X*. *ulophyllodes*, *XAu*) and soil were sampled at three sites with varied distances to a road in a semiarid sandland in Inner Mongolia, China and analyzed for concentrations of 42 elements to assess the contribution of soil input and road traffic to lichen element burdens, and to compare element concentration differences between the two lichens. The study showed that multielement patterns, Fe:Ti and rare earth element ratios were similar between the lichen and soil samples. Enrichment factors (EFs) showed that ten elements (Ca, Cd, Co, Cu, K, P, Pb, S, Sb, and Zn) were enriched in the lichens relative to the local soil. Concentrations of most elements were higher in *XAu* than in *XAa* regardless of sites, and increased with proximity to the road regardless of lichen species. These results suggested that lichen element compositions were highly affected by soil input and road traffic. The narrow-lobed sorediate species were more efficient in particulate entrapment than the broad-lobed nonsorediate species. *XAa* and *XAu* are good bioaccumulators for road pollution in desert and have similar spatial patterns of element concentrations for most elements as response to road traffic emissions and soil input.

## Introduction

Lichen bioaccumulation of atmospheric contaminants is a reliable tool for monitoring atmospheric element deposition^1–5^. Lichens are dependent on the atmosphere for nutrients, have great ability to entrap atmospheric contaminants due to the high surface/volume ratio and wide intercellular space, and have great tolerance to high concentrations of atmospheric pollutants^4,5^. This technique has been adopted as a complementary or an alternative method to traditional (instrumental) methods, which are costly and are just used to monitor limited number of pollutants (mainly CO, SO_X_, NO_X_, and dust)^6^.

Road traffic and dust deposition are two of the most serious sources of air pollution in China^6^, particularly in some desertified regions where dust storms and increasing road networks have emitted large amounts of contaminants in recent decades. Lichens have been used to monitor road traffic emissions in diverse ecosystems^7–12^, with only a few studies conducted in desert ecosystems, for example, the Sonoran Desert of USA^13^, the Negev Desert of Isreal^14–16^ and Chinese deserts^17,18^. These studies suggest that element compositions in desert lichens are highly affected by soil dust deposition and anthropogenic emissions. The studies conducted in Chinese deserts also suggest that different desert lichens accumulate elements in different amounts, but the lichen element concentrations respond similarly to road pollution levels^17,18^. In addition, the most concerned issues in lichen biomonitoring studies are to differentiate between the elements originating from road traffic emissions and those of geogenic origin and to assess the contribution of soil to the element burden of biomonitors. A comparison of multielement patterns, Fe:Ti ratios and the chondrite-normalized rare earth element (REE) parameters in biological and environmental samples are powerful tools for this purpose^19–28^.

**Figure 1:**
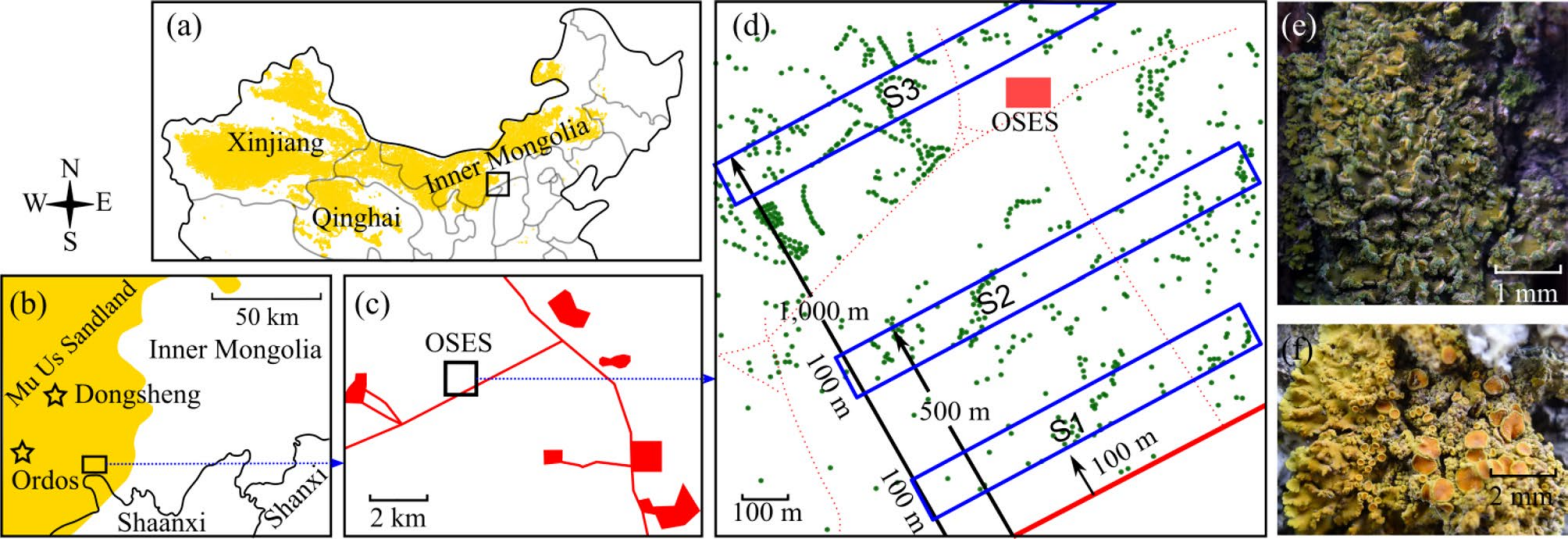
Experimental design. **(a)** Location of the study area in China; the golden areas indicate deserts. **(b)** Location of Mu Us Sandland and the study area; the stars indicate cities; the golden area indicates sandland. **(c)** Coal mines (red shadows) and industrial roads (red lines) near OSES (Ordos Sandland Ecosystem Station). **(d)** Three sampling sites (in blue brackets), trees (green dots), industrial road (red solid line) and private paths (red dotted lines). **(e)*** Xanthoria ulophyllodes*, showing the finely divided lobes with soredia. **(f)** *X. alfredii*, showing the foliose thallus and apothecium.

Ordos Sandland Ecological Station (OSES) is an ideal site for investigating the effects of soil deposition, road traffic emission and lichen species on lichen elemental compositions. OSES is a typical sandland ecosystem characterized by heavy sand-dust deposition, intense coal mining activity and severe vehicle traffic emission in recent decades in Inner Mongolia, China (Fig. 1a–d). In this ecosystem, *Xanthoria alfredii* (*XAa*) and *X. ulophyllodes* (*XAu*) are common lichens on trees close to a nearby industrial road (Fig. 1d). The two lichens are distinctive in morphology; *XAu* has finer lobes (< 0.5 mm wide) disintegrated into the ecorticated soredia at margins (Fig. 1e), while *XAa* has nonsorediate and broader lobes (0.5–1 mm wide; Fig. 1f).

In this research, we sampled *XAa*, *XAu* and surface soil in 3 sites with different distances from an industrial road (S1: 100–200 m from the road; S2: 400–500 m; S3: 900–1,000 m) in OSES (Fig. 1a–f). Elemental concentrations for a suite of 42 elements [Al, Ba, Ca, Cd, Co, Cs, Cu, Fe, K, Mg, Mn, Na, Nb, Ni, P, Pb, Rb, S, Sb, Sc, Sr, Th, Ti, Tl, U, V, Y, Zn, and 14 lanthanoids (La, Ce, Pr, Nd, Sm, Eu, Gd, Tb, Dy, Ho, Er, Tm, Yb, and Lu)] were quantified. The aims were to verify (1) if the contributions of soil and road traffic may enhance the lichen element concentration, and (2) if the element concentration differs between the two lichen species. The striking point of the present research lies in the fact that it is one of the few studies investigating the species-specific response of lichen element concentrations to road traffic in desertified regions.

## Results

### Concentrations and Fe:Ti ratios

Table 1 summarizes the analytical data of 42 elements and the Fe:Ti ratios in *XAa* (n = 20), *XAu* (n = 21) and soil (n = 9). All elements are normally distributed (p > 0.05; Shapiro–Wilk W test), except for 5 elements (Ce, Er, Ni, Pr, and Tb) in *XAu* with just slight deviations. The soil had lower concentration variations [coefficient variation (CV): 5.65–14.60%] than *XAa* (CV: 12.41–24.63%) and *XAu* (CV: 10.21–29.00%). The concentrations of all elements in the soil were not significantly different among the sites (independent samples t test, p > 0.05). The soil had higher concentrations than lichens for most elements in all sites (independent samples t test for each site, p ≤ 0.05; Table 1), in spite of some exceptions. These exceptions are as follows: (1) concentrations of 5 elements (Cd, P, S, Sb, and Zn) were higher in lichens than in soil in all sites; and (2) concentrations of Sb were similar between *XAa* and soil in S2 and S3.

**Table 1:** Statistic summary of element concentrations (data are in μg/g dry weight, CV in %) and Fe:Ti ratios in the lichen and soil samples from Mu Us Sandland. Statistic: “!” indicates higher values for the lichen than soil; “#” indicates similar values for lichens and soil in S2 and S3; otherwise, the values are lower in lichens than in soil (p ≤ 0.05, independent samples t test on raw concentrations and log10-normalized Fe:Ti ratios) in all sites.

	Lichens									Soil (n = 9)			
	*XAu* (n = 21)				*XAa* (n = 20)				*XAu*:*XAa*				
	Mean	CV (%)	Min	Max	Mean	CV (%)	Min	Max	Mean	Mean	CV (%)	Min	Max
Al	8136	11.78	6884	10,638	6694	14.47	5334	9000	1.21	63,640	6.29	57,531	69,560
Ba	115.0	12.71	93.75	150.4	87.43	15.93	63.45	122.4	1.30	546.6	6.13	488.6	604.6
Ca	3843	13.42	3053	5012	2911	15.56	2146	3865	1.32	7271	11.04	5993	8644
Cd	**0.947!**	16.62	0.726	1.307	**0.710!**	14.74	0.513	0.921	1.33	0.179	9.06	0.164	0.210
Ce	11.23	12.99	9.301	15.56	7.640	17.14	5.665	10.88	1.47	66.37	6.10	59.73	72.29
Co	4.330	11.76	3.299	5.375	4.058	13.73	3.303	5.174	1.06	12.10	7.74	10.12	13.14
Cs	1.637	15.97	1.251	2.307	1.460	13.89	1.176	1.856	1.12	6.329	10.57	4.935	7.154
Cu	12.87	14.56	9.870	17.98	9.459	15.20	7.205	13.27	1.35	20.55	7.97	17.30	22.25
Dy	0.885	22.06	0.644	1.378	0.538	22.28	0.374	0.838	1.62	4.794	7.98	4.166	5.279
Er	0.488	13.75	0.399	0.678	0.354	17.40	0.254	0.502	1.37	2.701	7.46	2.316	2.930
Eu	0.231	19.91	0.147	0.337	0.149	24.63	0.103	0.235	1.55	1.273	7.01	1.134	1.390
Fe	6361	13.44	5047	8787	5059	14.19	3772	6690	1.25	28,234	7.54	24,016	30,419
Gd	0.840	20.67	0.639	1.279	0.565	22.67	0.382	0.886	1.47	4.564	6.23	4.133	4.947
Ho	0.182	17.12	0.132	0.264	0.127	18.68	0.088	0.177	1.43	0.920	7.89	0.796	1.014
K	8213	10.21	6693	9317	7314	12.41	5916	9021	1.12	18,533	6.40	17,067	20,168
La	5.478	13.22	4.506	7.553	4.152	16.07	3.110	5.844	1.32	33.28	6.83	30.42	36.88
Lu	0.074	13.74	0.060	0.102	0.054	15.74	0.040	0.076	1.36	0.441	7.63	0.364	0.477
Mg	2505	14.90	1955	3439	2078	19.27	1465	3034	1.19	8875	14.60	6280	10,232
Mn	160.5	12.77	129.0	212.9	128.6	14.58	103.8	176.1	1.24	586.3	7.82	509.1	653.8
Na	2511	12.46	2043	3304	1953	16.56	1501	2731	1.28	9,787	10.35	8,854	11,948
Nb	1.403	18.38	1.088	2.074	1.184	12.71	0.912	1.484	1.19	14.009	7.56	12.08	15.50
Nd	5.647	13.19	4.631	7.744	3.833	16.55	2.892	5.448	1.47	28.03	5.99	25.66	30.29
Ni	8.269	12.47	6.883	11.34	6.392	14.59	5.029	8.488	1.29	28.85	9.08	23.66	31.96
P	**2493!**	11.80	2068	3135	**2217!**	13.75	1818	2991	1.12	402.5	7.05	360.8	461.9
Pb	14.93	11.99	12.04	20.19	11.51	14.73	8.760	15.25	1.29	22.92	9.69	18.49	25.24
Pr	1.474	18.32	1.142	2.174	0.963	18.21	0.709	1.416	1.52	7.575	6.53	6.932	8.453
Rb	16.16	11.96	13.31	20.80	15.08	13.55	11.52	19.32	1.07	91.43	8.27	81.04	103.2
S	**3271!**	11.39	2502	4013	**2710!**	13.06	2018	3312	1.20	348.0	8.27	314.9	412.4
Sb	**0.854!**	16.57	0.655	1.176	**0.643#**	16.71	0.454	0.861	1.34	0.597	6.17	0.551	0.649
Sc	1.803	15.52	1.404	2.521	1.395	16.55	1.036	1.980	1.29	10.72	7.36	9.651	11.75
Sm	0.930	15.67	0.700	1.307	0.647	20.54	0.470	0.963	1.44	5.344	6.15	4.792	5.835
Sr	38.44	16.09	29.42	55.50	33.08	14.27	24.66	42.54	1.16	197.3	6.70	174.5	219.2
Tb	0.157	29.00	0.114	0.322	0.093	20.31	0.064	0.139	1.66	0.785	5.75	0.706	0.834
Th	1.666	12.23	1.373	2.234	1.329	14.78	1.010	1.804	1.26	10.02	9.76	8.643	11.59
Ti	1124	13.42	902.0	1482	854.6	14.41	647.0	1167	1.31	4,798	5.65	4285	5146
Tl	0.203	20.17	0.136	0.289	0.171	16.64	0.128	0.211	1.20	0.645	7.80	0.567	0.754
Tm	0.074	12.99	0.059	0.103	0.053	16.87	0.038	0.075	1.39	0.405	8.44	0.335	0.440
U	0.236	13.51	0.187	0.314	0.175	14.77	0.138	0.232	1.34	1.853	8.39	1.620	2.047
V	15.28	13.67	12.32	21.18	11.75	16.86	8.160	16.41	1.30	96.96	6.08	87.37	108.1
Y	4.788	13.38	3.968	6.499	3.452	16.65	2.545	4.854	1.38	24.71	8.42	21.15	26.97
Yb	0.483	13.42	0.396	0.663	0.350	16.88	0.259	0.495	1.38	2.698	8.70	2.273	2.968
Zn	**156.2!**	16.36	113.52	214.0	**122.8!**	17.44	91.31	172.3	1.27	66.07	9.15	53.63	72.37
Fe:Ti	5.66	2.31	5.37	5.93	5.90	4.13	5.62	6.75		5.90	8.63	4.80	6.63

The values of *XAu*:*XAa* are calculated based on the mean values in each site.

*XAa, Xanthoria alfredii*; *XAu, X. ulophyllodes*.

The Fe:Ti ratios were not significantly different between *XAa* (5.90 ± 0.24) and soil (5.90 ± 0.51), and *XAu* (5.66 ± 0.13) and soil in all sites. All the ratios of *XAa*, *XAu* and soil did not show significant differences among the sites (p > 0.05; independent samples t test).

### Multielement patterns and EFs

**Figure 2:**
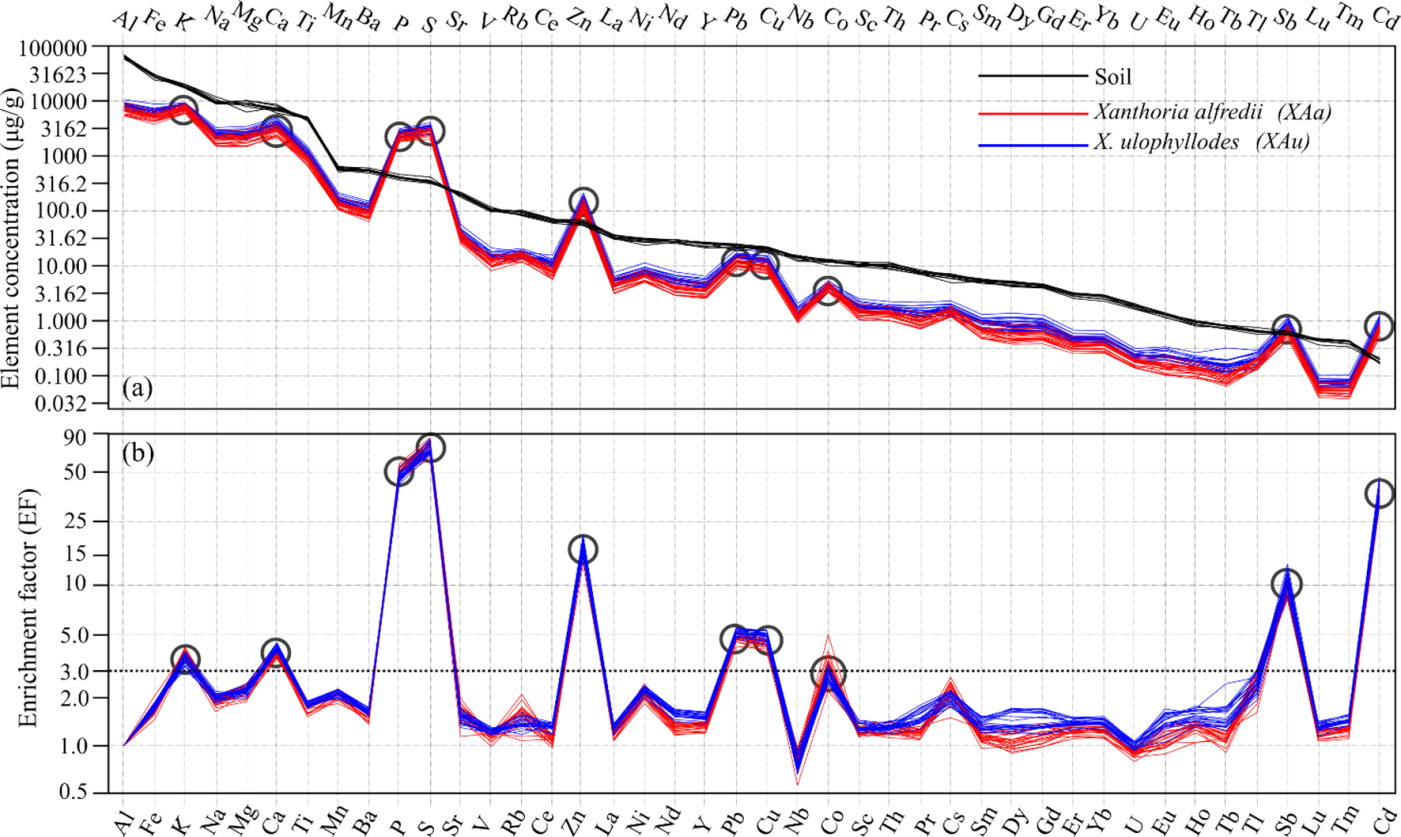
Multielement patterns and enrichment factors. **(a)** Multielement patterns of *Xanthoria alfredii*, *X. ulophyllodes* and local soil. **(b)** Enrichment factors for elements in lichens. Elements are arranged by decreasing concentration in the soil. The hollow circles indicate elements with an EF of > 3. *XAa*: n = 20. *XAu*: n = 21. Soil: n = 9.

Figure 2a shows that the multielement patterns of the soil samples are similar to one another, characterized by the decreasing concentration trends from Al to Cd. The same roughly holds true for the lichen samples (Fig. 2a), with the exception of 10 elements (K, Ca, P, S, Zn, Pb, Cu, Co, Sb, and Cd) which are enriched in lichens with respect to soil (EF > 3; Fig. 2b).

### REE patterns

**Figure 3:**
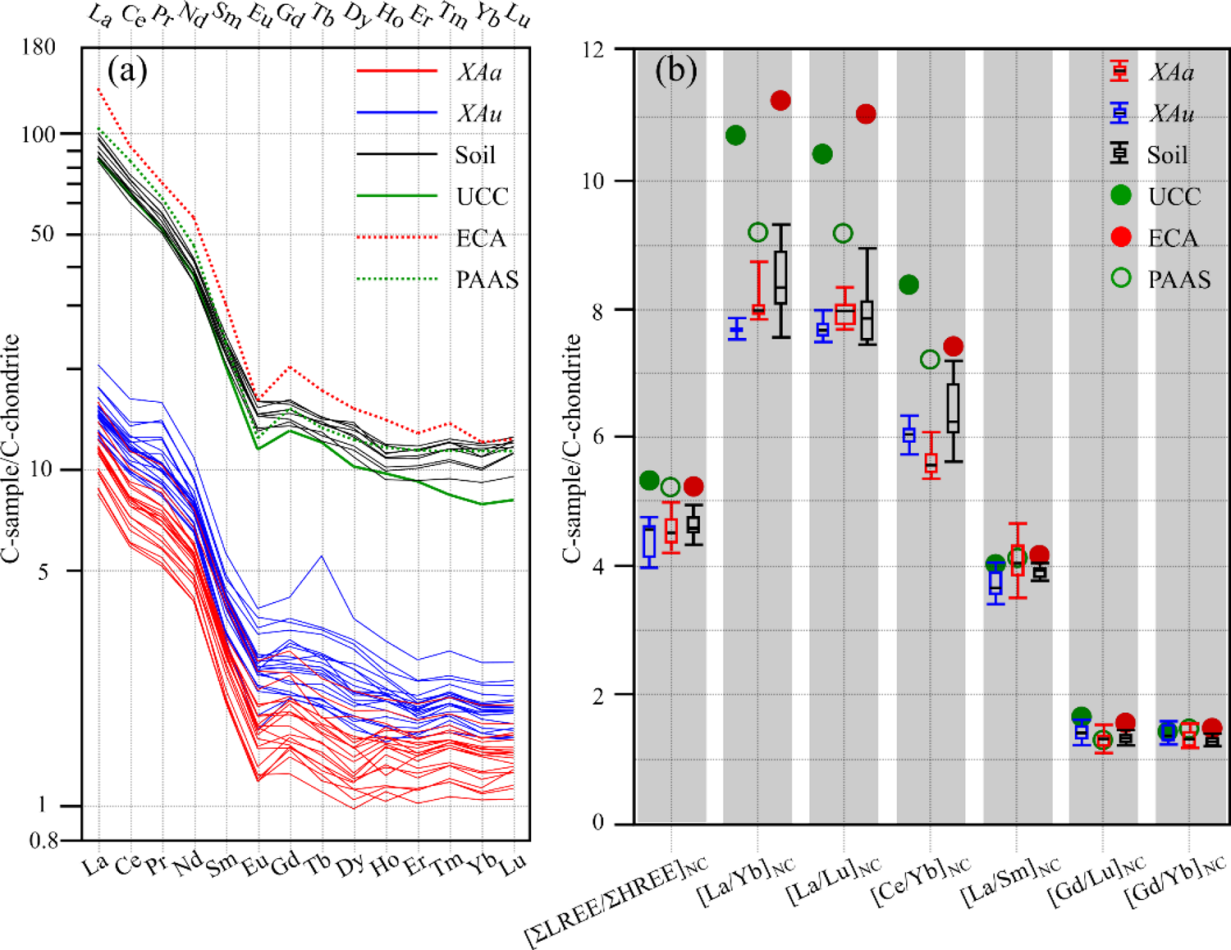
REE patterns of *XAa*, *XAu*, local soil, UCC, ECA and PAAS. **(a)** Chondrite-normalized REE patterns. **(b)** Boxplot of REE ratios normalized to chondrite for *XAa*, *XAu* and soil. *XAa*: n = 20. *XAu*: n = 21. Soil: n = 9. *C-sample *element concentration of the samples, *C-chondrite *element concentration of chondrite, *XAa—Xanthoria alfredii*, *XAu—X. ulophyllodes*, *ECA *argillaceous rocks in the eastern part of China, *PAAS* post-Archean Australian shale, *UCC* upper continental crust, *REE* rare earth element, *LREE* light REE (La, Ce, Pr, Nd, Sm, and Eu), *HREE* heavy REE (Gd, Tb, Dy, Ho, Er, Tm, Yb, and Lu).

Figure 3a shows the chondrite-normalized REE distribution patterns of the lichen and soil samples, upper continental crust (UCC), post-Archean Australian shale (PAAS) and argillaceous rocks in the eastern part of China (ECA). Despite great REE concentration differences, patterns of these samples are roughly similar to one another.

Figure 3b illustrates the chondrite-normalized parameters ([ΣLREE/ΣHREE]_NC_, [La/Yb]_NC_, [La/Lu]_NC_, [Ce/Yb]_NC_, [La/Sm]_NC_, [Gd/Yb]_NC_, and [Gd/Lu]_NC_) to evaluate the fractionation of light REEs (LREEs; La, Ce, Pr, Nd, Sm, and Eu) and heavy REEs (HREEs; Gd, Tb, Dy, Ho, Er, Tm, Yb, and Lu). The lichen and soil samples had similar values for all 7 parameters, among which 4 parameters ([ΣLREE/ΣHREE]_NC_, [La/Yb]_NC_, [La/Lu]_NC_, and [Ce/Yb]_NC_) are below the ranges of those in ECA, PAAS, and UCC.

### Correlation and differences

**Figure 4:**
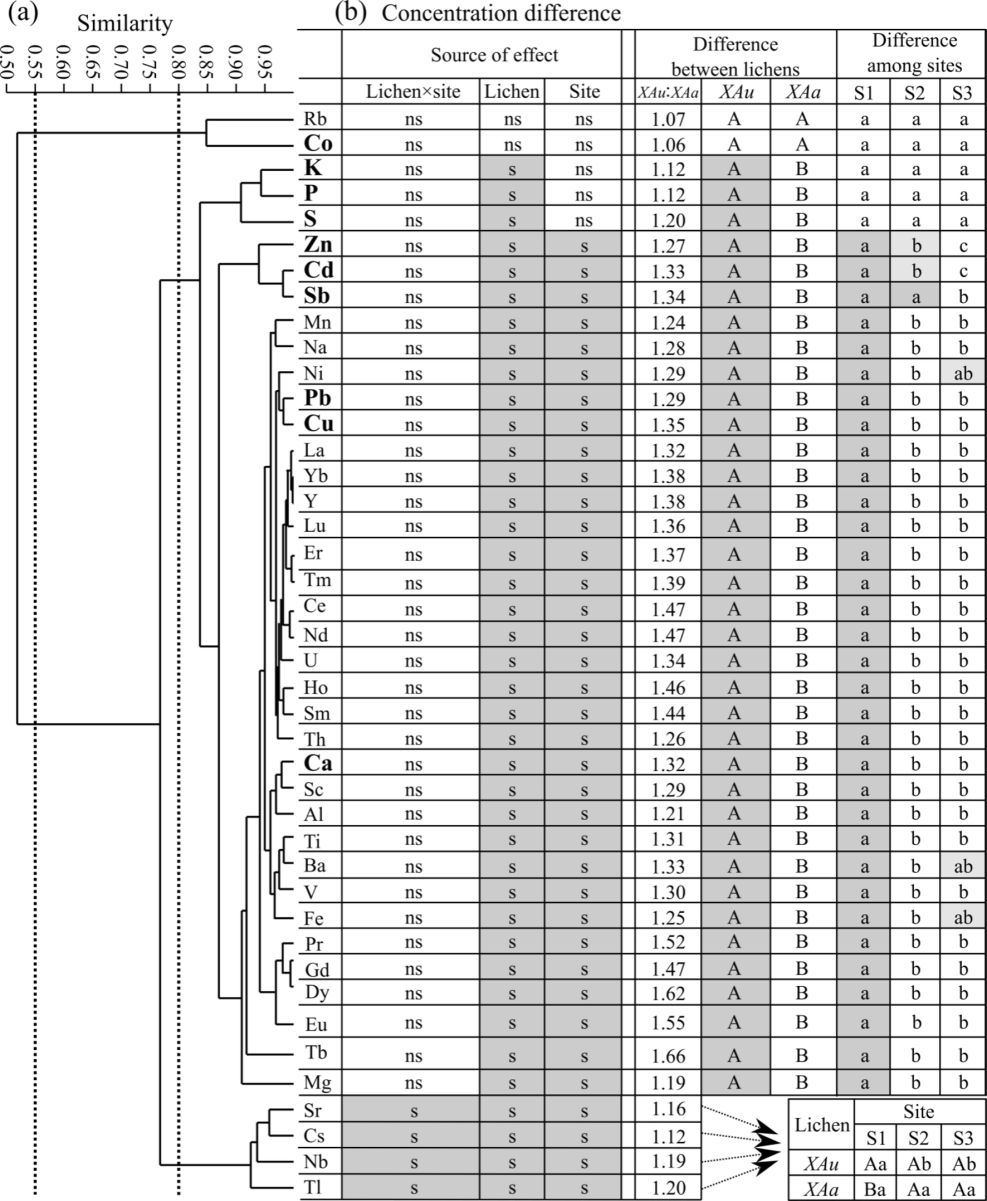
Results of cluster analysis and two-way ANOVA on lichens. **(a)** Dendrogram of UPGMA cluster analysis on a correlation matrix of z-score standardized concentrations. The dotted lines denote a correlation coefficient of 0.55 and 0.80. **(b)** Concentration differences between *XAa* (n = 20) and *XAu* (n = 21) and among sites (S1: 100–200 m from the road; S2: 400–500 m; S3: 900–1,000 m). Statistic: “s” and “ns” denote the significant effects and nonsignificant effects at α = 0.05, respectively. Different capitalized letters denote the significant differences in concentration between *XAa* and *XAu*. Different small letters denote the significant differences in concentration among sites. Elements in bold are enriched elements with a mean EF of > 3.0 (Fig. 2b). *XAa—Xanthoria alfredii*, *XAu—X. ulophyllodes*.

Figure 4a shows the results of UPGMA cluster analysis on a correlation matrix of 42 elements in the lichen samples. Figure 4b shows the results of two-way ANOVA on z-score standardized concentrations of 42 elements, with lichen species and sites as the fixed factors. The cluster analysis represents most of the concentration correlations between elements (cophenetic correlation coefficient = 0.970). All elements show a good positive correlation with one another at a correlation similarity of 0.50.

Rb and Co are separated from the other elements at a correlation similarity of 0.55 (Fig. 4a). Concentrations of both elements are similar between lichen species and among sites (*XAu*:*XAa* = 1.06–1.07; *p* > 0.05 for the main and interaction effects; Fig. 4b).

Cs, Nb, Sr, and Tl are separated from the other elements at a correlation similarity of 0.80 (Fig. 4a). There is a significant interaction effect of the lichen species and sites on concentrations of these metals (Fig. 4b). The concentrations of these metals tend to decrease with distance to the road in *XAu* but are nearly identical among the sites in *XAa*; *XAu* is higher than *XAa* at site S1, but *XAu* and *XAa* are not significantly different at sites S2 and S3 (Fig. 4b).

The remaining 36 elements form a cluster at a correlation similarity matrix of > 0.80 (Fig. 4a). There is no significant interaction effect of the lichen species and sites on the concentrations of these metals (Fig. 4b). Concentrations of these elements are higher in *XAu* than in *XAa* (*XAu*:*XAa* = 1.12–1.66) and are higher in site S1 than in S2 and/or S3, with the exception of 3 elements (K, P, and S), of which the concentrations are not significantly different between the sites (Fig. 4b).

## Discussion

### Deposition degree

OSES can be considered a fairly contaminated place when comparing the lichen data with those of epiphytic lichens in other studies. The concentrations of most elements in the lichen samples are higher than or similar to those in epiphytic lichens from the desertified sites or sites near roads (Supplementary Table S1), such as similar ecosystems in Xilinhot, Inner Mongolia, China^17,18^. This finding is also the case when the data in this study are compared with the data from epiphytic lichens near roads in Turkey^9,29^, India^30^, and France^31^ (Supplementary Table S1). However, our data of most elements are lower than or at the lower range of 26 elements in *Flavopunctelia soredica* transplanted along the two busy roads in a highly polluted area of Hebei, China^7^ (Supplementary Table S1).

### Soil contribution

Thirty-two elements (Al, Ba, Ce, Cs, Dy, Er, Eu, Fe, Gd, Ho, La, Lu, Mg, Mn, Na, Nb, Nd, Ni, Pr, Rb, Sc, Sm, Sr, Tb, Th, Ti, Tl, Tm, U, V, Y, and Yb) in *XAa* and *XAu* are highly affected by soil input. These elements show similar multielement patterns between the lichen and soil samples (Fig. 2a) and have EFs of < 3.0 (Fig. 2b). An EF of < 3.0 suggests crustal input^19,32^. In this ecosystem, the vegetation is sparse, and the soil is vulnerable to wind erosion. Most of these elements, such as Al, Fe, Rb, Sc, Ti and lanthanoids are attributed to windblown dust input in similar ecosystems of Inner Mongolia^17,18^. Fe:Ti ratios are similar among *XAa* (5.90 ± 0.24), *XAu* (5.66 ± 0.13), and soil (5.90 ± 0.51; Table 1) in all sites, suggesting the trapping of local soil particulates in lichen thalli^26,27^. In a similar ecosystem of Inner Mongolia, the similar Fe:Ti ratios between the epiphytic foliose lichens (*Phaeophyscia hirtuosa* and *XAa*; 12.30–13.12) and the local soil samples (12.27) are attributed to an entrapment of windblown soil particulates in lichen thalli^18^.

The high soil contribution is also supported by the REE patterns (Fig. 3). The lichen and soil samples, UCC, PAAS and ECA have roughly similar REE distribution patterns (Fig. 3a). The 4 parameters ([ΣLREE/ΣHREE]_NC_, [La/Yb]_NC_, [La/Lu]_NC_, and [Ce/Yb]_NC_) in lichen samples are lower than those in UCC, ECA and PAAS, but are similar to those in local soil (Fig. 3b), indicating that the REE composition in the lichen samples is highly related to local soil. This conclusion agrees with the results of other studies: the REE accumulation in mosses and lichens is attributed to soil dust deposition^20,21,28,33,34^.

The soil contribution to lichen element burdens might be marked by redeposition of local soil contaminants from human activities such as coal mining and transport. The road traffic effect is evident in data of the 22 elements (the 14 lanthanoids, Al, Na, Ni, Sc, Th, U, V, and Y), which are closely correlated (Fig. 4a) and have the highest concentrations at the site close to the road (S1) regardless of lichen species (Fig. 4b). This spatial pattern is also the case for the other 4 elements (Sr, Nb, Cs, and Tl) observed in *XAu* (Fig. 4b). Other lichen biomonitoring studies conducted close to roads also have found similar distance-dependent concentration patterns attributed to the enhancement of the deposition/redeposition of soil dust by traffic^7,12,18,30^.

### Enriched elements and road traffic effects

The results of EFs (Fig. 2b) show that ten elements (Ca, Cd, Co, Cu, K, P, Pb, S, Sb, and Zn) are enriched in *XAa* and *XAu* relative to the local soil. An EF of > 3.0 is an indicator of anthropogenic and/or nonlocal sources or bioregulation of these elements in lichen thallus^19,32^.

Five enriched metals (Cd, Cu, Pb, Sb, and Zn) are likely to have come from traffic emissions. These metals are typical traffic-related pollutants emitted through fossil fuel combustion, fuel additives, tire and brake pad abrasion, corrosion, and lubricating oils^4,6^. These metals have the highest concentrations at S1 and lowest concentrations at S3, regardless of lichen species (Fig. 4b). In Negev deserts, the amount of Pb in lichens has been found higher at one site close to a road than at other sites^14^. The higher concentrations of these metals in lichens close to roads or at sites with high traffic levels have also been reported in other studies^7–10,30,35–37^.

The concentrations of 4 enriched elements (Co, K, P, and S) did not undergo any changes with distance from the road regardless of lichen species (Fig. 4b). One explanation for the spatial pattern of S may be the impact of the coal emissions. Sulfur is rich in coals and is an important contaminant during coal combustion in China^6^. Sulfur emissions from several coal mines around the study site may represent a significant source of S in lichens and surface soils. Bioregulation of these essential nutrients in lichen thallus may also be responsible for this pattern. In fact, the trends of nutrients are often different from or even inverse to those of pollutants in lichens. For example, concentrations of traffic-related heavy metals increase with proximity to the road, while some nutrients (K, P and Mn) show a reverse trend due to nutrient leakage as a result of road pollution^8^. The metals (Cu, Pb, and Zn) in *Xanthoparmelia scabrosa* decrease from urban to rural areas, whereas three nutrients (K, P, and S) show an inverse pattern^38^.

Calcium appears to come from traffic-related dust redeposition superimposed on local soil deposition. The spatial pattern for Ca is similar to typical soil-derived metals such as Ti and Sc (Fig. 4a, b). This metal is seldom released by vehicle emissions. The enrichment of Ca in *XAa* and *XAu* (Fig. 2b) may be due to the preferential absorption/retention of this nutrient by lichens. Calcium can accumulate greatly in lichens^27,39^ in the form of insoluble organic calcium salt such as calcium oxalate.

### Lichen species differences

The research results show a species- and element- specific accumulation of elements in lichens. The narrow-lobed sorediate lichen *XAu* (Fig. 1e) has a higher accumulation capability for 40 elements (all elements barring Rb and Co; Fig. 4b) than the broad-lobed nonsorediate lichen *XAa* (Fig. 1f). These results are in accordance with the other studies suggesting that the presence of soredia and narrower lobes allows a higher surface/volume ratio to enhance the capability of the entrapment and retention of atmospheric particles^1–4,40^. The degree of concentration difference between *XAu* and *XAa* is highest for the 14 lanthanoids (*XAu*:*XAa*: 1.32–1.66) and lowest for the 5 elements (Co, Cs, K, P, and Rb; *XAu*:*XAa*: 1.06 ~ 1.12; Table 1, Fig. 4b). Other studies also reported that different lichens accumulate different elements to different extent^2^.

Despite the species- and element-specific contrasts in element concentrations, *XAa* and *XAu* share similar multielement patterns (Fig. 2a), EFs (Fig. 2b) and REE patterns (Fig. 3), and show similar concentration trends with the variation of distance from the road for most elements (Fig. 4b). These results are consistent with those of other studies reporting that the element concentration differences among lichen species mainly manifest different accumulation rates, but the spatial/temporal trends of individual elements remain similar^7,8,21,26^.

## Conclusions

The element compositions in *XAa* and *XAu* are highly affected by road traffic and local soil. Five metals (Cd, Cu, Pb, Sb, and Zn) accumulated in lichens can be traced to traffic emissions. Local soil input has great influence on the concentrations of 33 elements (Al, Ba, Ca, Ce, Cs, Dy, Er, Eu, Fe, Gd, Ho, La, Lu, Mg, Mn, Na, Nb, Nd, Ni, Pr, Rb, Sc, Sm, Sr, Tb, Th, Ti, Tl, Tm, U, V, Y, and Yb) in lichen thalli and their content reaches highest in the places close to the roads due to the redeposition of road dust. Concentrations of 4 nutrients (Co, K, P, and S) in *XAa* and *XAu* show little changes with proximity to the road, possibly due to the interaction between lichen physiology and air pollution. Concentrations of the most elements are higher in *XAu* than those in *XAa*. The two lichens can serve as bioaccumulators to monitor atmospheric element deposition near roads in deserts and yield similar spatial patterns of element concentrations in most cases.

## Methods

### Investigation area

Ordos Sandland Ecological Station (N 39°29′, E 110°11′; OSES, Institute of Botany, Chinese Academy of Sciences) is located at Mu Us Sandland, southeastern Ordos Plateau, Inner Mongolia, China (Fig. 1a, b). This area has a semiarid monsoon climate with a mean annual evaporation of 2093 mm. The mean annual precipitation is 350–380 mm, largely (60–80%) in the form of rainfall during June to August. The elevation is approximately 1290 m a.s.l. The soil is sandy loam and aeolian sandy soil. The region has been severely desertified due to overgrazing, mining and other anthropogenic activities and is one of the most important sources of sand dust storms in China. In 2013, the landscape was characterized by semifixed and moving sand dunes with patches of cultivated trees (mainly *Poplus* spp.), psammophytic shrubs and herbs.

The station lies at a rural site approximately 35 km from the nearest city (Fig. 1b). However, the station is surrounded with several coal mines and mine tailings and is adjacent to industrial roads for coal transportation (Fig. 1c). The nearest coal mine is 3 km away and its operation commenced in Dec 2009. About a dozen workers and students stayed at the station mainly from late May to early October. There were some private paths with few, if any, vehicles (Fig. 1d).

### Sample collection

*XAa*, *XAu* and soil were sampled during 8–10 August 2013. To investigate the road traffic effects on lichen element burdens, three sites of 100 × 800–1000 m each were selected at an increasing distance from the road: S1 (100–200 m from the road), S2 (400–500 m), and S3 (900–1000 m), with the longest side parallel to the industrial road. The area within 100 m of the road was not included because there were very few trees and epiphytic lichens (Fig. 1d).

In each site, 6–8 homogeneous plots [i.e., the plots had Poplar trees with uniform density, similar stem diameter (15–20 cm) and abundant lichen individuals], each with an area of 5–8 × 5–8 m, were selected for each of the two lichens. Each plot was represented by a single composite sample made up of 15–25 thalli (6–10 g dw) randomly collected from all aspects of 3–5 Poplar trees at a height of approximately 1.0–2.0 m from the ground by using a knife. An influence of inter-individual differences in size, age, or microclimatic factors on lichen element concentrations is nonnegligible^31^. Thus the large composite samples has been frequently adopted in the biomonitoring studies to reduce the effects of sample/habitat heterogeneity^28,41,42^. Due to the complex vegetation conditions and the high dependency of plot selection on the availability of trees and lichens, the experiment is an unbalanced design with unequal number of samples for *XAa* and *XAu* in each site. For most cases, *XAa* and *XAu* were collected from different plots. A total of 41 composite samples were collected, with 20 for *XAa* and 21 for *XAu* (Table 2).

**Table 2:** Sample size of *XAa*, *XAu*, and soil. Each lichen sample is composed of 15–25 thalli from a plot.

Site (distance to the road)	Lichen species			Soil
	*XAa*	*XAu*	Total	
S1 (100–200 m)	6	7	13	3
S2 (400–500 m)	8	7	15	3
S3 (900–1000 m)	6	7	13	3
Total	20	21	41	9

Three samples of approximately 100 g of shallow (5 cm deep) neighboring soil, each composed of five subsamples, were also randomly collected in each site. All samples were placed in plastic bags to prevent contamination and were taken to the laboratory for later identification and analysis.

### Sample preparation and measurement

Apothecia of *XAa* were removed manually. All samples were carefully cleaned under a low-powered stereomicroscope, dried in oven to a constant weight at 60 °C for 72 h, ground and homogenized in a grinding mill equipped with tungsten carbide jars (Retsch MM400; Retsch GmbH, Haan, Germany). Aliquots of 200–300 mg of each homogenized sample were mineralized in a mixture of HNO_3_ and H_2_O_2_ for lichens, and in a mixture of HCl, HNO_3_, HF and HClO_4_ for soil. The concentrations of 42 elements (Al, Ba, Ca, Cd, Ce, Co, Cs, Cu, Dy, Er, Eu, Fe, Gd, Ho, K, La, Lu, Mg, Mn, Na, Nb, Nd, Ni, P, Pb, Pr, Rb, S, Sb, Sc, Sm, Sr, Tb, Th, Ti, Tl, Tm, U, V, Y, Yb, and Zn) were determined on a dry weight basis using an inductively coupled plasma mass spectrometer (ICP-MS; Agilent 7700X; Agilent Technologies, Tokyo, Japan) at the Hebei Research Center for Geoanalysis.

Analytical quality control of the ICP-MS results was assured by using a series of standard reference materials: GBW10014 cabbage, GBW10015 spinach, GBW10052 green tea and IAEA-336 Portuguese lichen. The results were within certified and/or suggested values. The analytical precision and accuracy are generally < 10%. These methods have been described in detail elsewhere^43^.

### Data treatment

The following chondrite-normalized ratios are reliable tools for evaluating REE fractionation in moss, lichen and substrate samples^20,28,33^. The [ΣLREE/ΣHREE]_NC_, [La/Yb]_NC_, [La/Lu]_NC_, and [Ce/Yb]_NC_ ratios are measures of fractionation between LREE and HREEs. The [La/Sm]_NC_ ratio is used to evaluate the LREE fractionation degree; and the [Gd/Yb]_NC_ and [Gd/Lu]_NC_ ratios, the HREE fractionation degree. These ratios are calculated according to Eq. (1):

1 $$ \left[ {{\text{A}}/{\text{B}}} \right]_{{{\text{NC}}}} = \, \left( {{\text{A}}_{{{\text{sample}}}} /{\text{A}}_{{{\text{chondrite}}}} } \right)/\left( {{\text{B}}_{{{\text{sample}}}} /{\text{B}}_{{{\text{chondrite}}}} } \right) $$

where A and B are the elements in question, the subscript “NC” indicates that the samples are normalized to the chondrite values^44^, and the subscripts “sample” and “chondrite” indicate which medium the concentration refers to.

The average values of the upper continental crust (UCC)^45^, post-Archean Australian shale (PAAS)^44^ and argillaceous rocks in the eastern part of China (ECA)^46^ are used for comparison in the study of REE distribution and fractionation.

The enrichment factor (EF) is calculated according to Eq. (2):

2 $$ {\text{EF}}_{{\text{X}}} = \, \left( {{\text{X}}_{{{\text{lichen}}}} /{\text{Al}}_{{{\text{lichen}}}} } \right)/\left( {{\text{X}}_{{{\text{soil}}}} /{\text{Al}}_{{{\text{soil}}}} } \right) $$

where X is the element in question, Al is the reference crustal element, and the subscripts “lichen” or “soil” indicate which medium the concentration refers to.

### Statistical analyses

Concentrations of each element are tested for normality using Shapiro–Wilk’s test and for homogeneity of variance using Levene’s test (α = 0.05). For each of the three sites, an independent samples t test is conducted to check whether the element concentration and Fe:Ti ratio (log10-transformed) in the soil are significantly different between sites and significantly different from those in *XAa* and in *XAu* (α = 0.05).

The raw concentrations of the lichen combined dataset and the soil samples are z-score standardized [(x-mean)/SD] respectively for subsequent analyses. A cluster analysis is conducted with the unweighted pair-group method plus arithmetic means (UPGMA) linkage method based on the correlation distance as a measure of similarity. A two-way analysis of variance (ANOVA) is performed to test the main and interactive effects of the lichen species (fixed factor of two levels, either *XAa* or *XAu*) and sites (fixed factor of three levels: either S1, S2, or S3) on each element (α = 0.05). A Tukey’s honestly significant difference (HSD) test is conducted for post hoc comparisons. Harmonic means are used in this analysis to correct the variations in sample size. A simple effect analysis is conducted in the case of significant interactive effects. All statistical analyses are performed using PAST 3.26 software (Ø. Hammer, April 2019). Plots are generated using PAST 3.26 software and Inkscape 0.92 software (Free Software Foundation Inc., USA).

## Supplementary information

Supplementary Information.
