# Supplementary material for: Two lichens differing in element concentrations have similar spatial patterns of element concentrations responding to road traffic and soil input
Source: Sci Rep. 2020 Nov 4;10:19001. doi: 10.1038/s41598-020-76099-x (PMC7642413; doi:10.1038/s41598-020-76099-x)
Supplement: Supplementary file 1 — Supplementary Information. [file 41598_2020_76099_MOESM1_ESM.docx]

Supplementary Information

Two lichens differing in element concentrations have similar spatial patterns of element concentrations responding to road traffic and soil input

Yuan-yuan Wu^1+^, Jing Gao^1+^, Guo-zhan Zhang^3^, Run-kang Zhao^2^, Ai-qin Liu^2^, Lian-wei Sun^2^, Xing Li^2^, Hong-liang Tang^1^，Liang-cheng Zhao^2^, Xiu-ping Guo^2^*, and Hua-jie Liu^1^*

^1^ School of Life Sciences, Institute of Life Science and Green Development, Hebei University, Baoding, Hebei 071002, China

^2^ Hebei Research Center for Geoanalysis, Baoding, Hebei 071051, China

^3^ Hebei Baoding Municipal Drainage Corporation, Baoding, Hebei 071051, China

* H.-J. L. (liuhuajie@foxmail.com), X.-P. G. (316489636@qq.com)

+ These authors contributed equally to this work

Content: Table S1

## Table S1. Element concentrations measured in epiphytic lichens from selected locations. Abbreviations: *CAf – Candelaria fibrosa*, *EVp – Evernia prunastri*, *FLs* – *Flavopunctelia soredica*, *PHad – Physcia adscendens*, *PHai* *– Physcia aipolia*, *PYc – Pyxine cocoes*, *XAa* *–* *Xanthoria alfredii*, *XAp – X*. *parietina*, *XAu – X. ulophyllodes*.

| Location | Turkey | Turkey | India | France | Inner Mongolia | | Hebei | Present study | |
| --- | --- | --- | --- | --- | --- | --- | --- | --- | --- |
| Site description | Polluted^29^ | Polluted^9^ | Polluted^30^ | Polluted^31^ | Desert^17^ | Desert^18^ | Polluted^7^ | Sandland | |
| Contaminant source | various | various | road | road | desert | road | road | road | road |
| Species | *XAp* | *PHad* | *PYc* | *EVp* | *PHai* | *CAf* | *FLs* | *XAu* | *XAa* |
| Statistics | Min.-Max. | Means | Means | Means | Mean | Mean | Min.-Max. | Min.-Max. | Min.-Max. |
| Al |  | 482.9–5541 | 886.2–1376 |  | 6998 |  | 5083–21928 | 6884–10638 | 5334–9000 |
| Ba |  |  |  |  | 116.5 | 81.19 | 71.39–371.5 | 93.75–150.4 | 63.45–122.4 |
| Ca |  |  |  |  |  | 2840 | 3265–17472 | 3053–5012 | 2146–3865 |
| Cd | 0.07–1.9 | 0.18–5.59 | 0.9–4.5 | 1.1 | 0.28 | 0.4 | 0.583–1.563 | 0.726–1.307 | 0.513–0.921 |
| Ce |  |  |  |  | 8.58 |  |  | 9.301–15.56 | 5.665–10.88 |
| Co | 1.56–30.62 |  |  | 0.7 |  | 2.35 | 1.180–5.054 | 3.299–5.375 | 3.303–5.174 |
| Cs |  |  |  |  | 1.29 |  | 0.964–3.349 | 1.251–2.307 | 1.176–1.856 |
| Cu | 2.95–32.52 |  | 6.3–10.5 | 13.2–23.2 | 9.52 | 16.91 | 13.90–86.16 | 9.87–17.98 | 7.205–13.27 |
| Fe | 1404–12459 | 521–5265 | 419–730 |  | 5567 | 7604 | 4464–21870 | 5047–8787 | 3772–6690 |
| K |  |  |  |  | 11870 | 8397 | 4878–10633 | 6693–9317 | 5916–9021 |
| La |  |  |  |  | 4.29 |  | 1.20–17.41 | 4.506–7.553 | 3.11–5.844 |
| Mg |  |  |  |  |  | 338.6 | 861–6323 | 1955–3439 | 1465–3034 |
| Mn | 7.31–354.7 | 0.74–91.63 |  |  | 135.7 | 269.9 | 142.9–914.4 | 129–212.9 | 103.8–176.1 |
| Na |  |  |  |  | 1437 | 1547 | 864.4–4946 | 2043–3304 | 1501–2731 |
| Ni |  | 5.59–22.96 |  |  | 5.65 | 10.1 | 5.472–33.06 | 6.883–11.34 | 5.029–8.488 |
| P |  |  |  |  | 2511 | 1419 |  | 2068–3135 | 1818–2991 |
| Pb | 0.4–39.41 | 0.16–47.25 | 19.4–89.1 | 28.1–160.9 | 8.2 | 10.78 | 9.392–120.2 | 12.04–20.19 | 8.76–15.25 |
| Rb |  |  |  | 4.1–12.8 |  |  | 4.571–39.03 | 13.31–20.8 | 11.52–19.32 |
| S |  |  |  |  |  |  | 2918–9416 | 2502–4013 | 2018–3312 |
| Sb |  |  |  | 3.7–3.7 | 0.36 | 0.41 | 0.552–2.952 | 0.655–1.176 | 0.454–0.861 |
| Sc |  |  |  |  | 0.95 |  |  | 1.404–2.521 | 1.036–1.980 |
| Sm |  |  |  |  | 0.67 |  | 0.338–2.211 | 0.7–1.307 | 0.47–0.963 |
| Sr |  |  |  |  |  | 36.01 | 23.63–99.84 | 29.42–55.50 | 24.66–42.54 |
| Tb |  |  |  |  | 0.09 |  | 0.06–0.275 | 0.114–0.322 | 0.064–0.139 |
| Th |  |  |  |  | 1.29 |  | 0.853–4.078 | 1.373–2.234 | 1.01–1.804 |
| Ti |  |  |  | 32.5–124.3 | 530.8 | 579.8 | 267.3–990.5 | 902.0–1482 | 647.0–1167 |
| Tl |  |  |  |  | 0.17 |  |  | 0.136–0.289 | 0.128–0.211 |
| U |  |  |  |  | 12.41 |  |  | 0.187–0.314 | 0.138–0.232 |
| V |  | 1.29–17.77 |  | 1.1–5.8 |  | 15.45 | 10.4–45.4 | 12.32–21.18 | 8.160–16.41 |
| Zn | 16.5–142.6 | 4.94–228.6 | 55.7–118.1 | 43.9–102.5 | 72.74 | 124 | 118.8–309.7 | 113.5–214.0 | 91.31–172.3 |

References

**7.** Zhao, L. L. *et al.* Element bioaccumulation in lichens transplanted along two roads: The source and integration time of elements. *Ecol. Indic.* **99**, 101–107, DOI: https://doi.org/10.1016/j.ecolind.2018.12.020 (2019).

**9.** Kurnaz, K. & Cobanoglu, G. Biomonitoring of air quality in Istanbul Metropolitan Territory with epiphytic lichen *Physcia adscendens* (Fr.) H. Olivier. *Fresen. Environ. Bull.* **26**, 7296–7308 (2017).

**17.** Liu, H. J. *et al.* Lichen elemental composition distinguishes anthropogenic emissions from dust storm inputs and differs among species: Evidence from Xilinhot, Inner Mongolia, China. *Sci. Rep.-UK.* **6**, 34694, DOI: https://doi.org/10.1038/srep34694 (2016).

**18.** Liu, H. J. *et al.* Elemental compositions of lichens from Duolun County, Inner Mongolia, China: Origin, road effect and species difference. *Sci. Rep.-UK.* **7**, 5598, DOI: https://doi.org/10.1038/s41598-017-06027-z (2017).

**29.** Hanedar, A. Assessment of airborne heavy metal pollution in soil and lichen in the Meric-Ergene Basin, Turkey. *Environ. Technol.* **36**, 2588–2602, DOI: https://doi.org/10.1080/09593330.2015.1039071 (2015).

**30.** Bajpai, R. & Upreti, D. Accumulation and toxic effect of arsenic and other heavy metals in a contaminated area of West Bengal, India, in the lichen *Pyxine cocoes* (Sw.) Nyl. *Ecotox. Environ. Safe.* **83**, 63–70, DOI: https://doi.org/10.1016/j.ecoenv.2012.06.001 (2012).

**31.** Ayrault, S., Clochiatti, R., Carrot, F., Daudin, L. & Bennett, J. P. Factors to consider for trace element deposition biomonitoring surveys with lichen transplants. *Sci. Total Environ.* **372**, 717–727, DOI: https://doi.org/10.1016/j.scitotenv.2006.10.032 (2007).
